# Cues, context, and long-term memory: the role of the retrosplenial cortex in spatial cognition

**DOI:** 10.3389/fnhum.2014.00586

**Published:** 2014-08-05

**Authors:** Adam M. P. Miller, Lindsey C. Vedder, L. Matthew Law, David M. Smith

**Affiliations:** Department of Psychology, Cornell UniversityIthaca, NY, USA

**Keywords:** retrosplenial cortex, hippocampus, context, navigation, long-term memory, learning, allocentric, consolidation

## Abstract

Spatial navigation requires memory representations of landmarks and other navigation cues. The retrosplenial cortex (RSC) is anatomically positioned between limbic areas important for memory formation, such as the hippocampus (HPC) and the anterior thalamus, and cortical regions along the dorsal stream known to contribute importantly to long-term spatial representation, such as the posterior parietal cortex. Damage to the RSC severely impairs allocentric representations of the environment, including the ability to derive navigational information from landmarks. The specific deficits seen in tests of human and rodent navigation suggest that the RSC supports allocentric representation by processing the stable features of the environment and the spatial relationships among them. In addition to spatial cognition, the RSC plays a key role in contextual and episodic memory. The RSC also contributes importantly to the acquisition and consolidation of long-term spatial and contextual memory through its interactions with the HPC. Within this framework, the RSC plays a dual role as part of the feedforward network providing sensory and mnemonic input to the HPC and as a target of the hippocampal-dependent systems consolidation of long-term memory.

## Introduction

Navigating within a familiar environment requires a neural representation of the navigation cues that define the environment and the spatial relationships among them. For example, traveling between two locations requires recognizing your current position, selecting a destination from memory, and remembering how these two locations fit within the broader navigation context. Although subjects sometimes navigate using a response strategy (e.g., turn right at the library, turn left at the bell tower), they are also capable of using knowledge about the spatial layout of the environment in order to navigate flexibly toward a goal (i.e., a cognitive map; Tolman, 1948)—an ability that is supported by interactions between the hippocampus (HPC), the anterior thalamic nuclei (ATN), and a network of cortical regions including the parahippocampal cortex, retrosplenial cortex (RSC), and posterior parietal cortex (O’Keefe and Dostrovsky, 1971; Moser et al., 2008; Calton and Taube, 2009; Vann et al., 2009). Spatial representation is closely related to other forms of memory processing including context processing and episodic memory due to a common reliance on representations of objects and object relationships (Eichenbaum et al., 1999). Indeed, many of the brain regions known to be involved in spatial navigation also play a prominent role in these other forms of memory processing. Among these regions, the HPC has a well-known role in the formation of new memories and the consolidation of long-term memories into the cortex (McClelland et al., 1995; Squire, 2004; Morris, 2006; Dudai, 2012). In many cases, long-term representations consolidated in cortical regions can then support spatial navigation independent of the HPC (Day et al., 2003; Winocur et al., 2005b; Maguire et al., 2006; Tse et al., 2007; but see Winocur et al., 2005a). Long-term memory stored in cortex also plays an important role in support of context representations (Bar and Aminoff, 2003) and episodic memory (Danker and Anderson, 2010). Here we review evidence that the RSC is positioned between regions that play an important role in memory formation and allocentric representation, and regions known to be involved in long-term memory and egocentric representation. We argue that the RSC plays a critical role in the formation and long-term storage of allocentric spatial representations by virtue of a more basic contribution processing cues and cue associations. These associations are essential building blocks of spatial and contextual representations, and also play an important role in episodic memory functions.

## Functional anatomy

**Figure 1:**
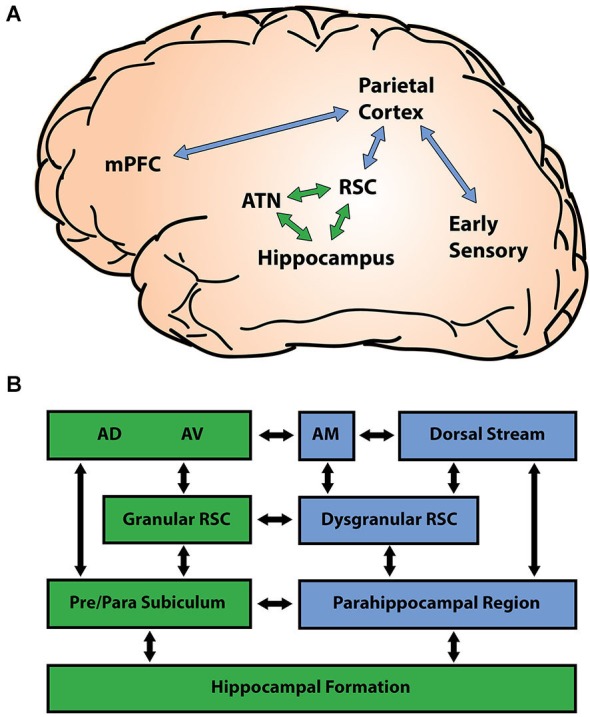
**Retrosplenial connectivity**. **(A)** The retrosplenial cortex (RSC) is centrally positioned between cortical sensory regions (blue) and limbic memory regions (green). The parietal lobe merges information arriving from early sensory areas, and shares connectivity with both the medial prefrontal cortex (mPFC) and the RSC. Reciprocal connections between the RSC, anterior thalamic nuclei (ATN), and the hippocampus (HPC) constitute a limbic memory circuit that is essential for many forms of learning and memory. **(B)** The RSC shows regional differences in connectivity with limbic (green) and cortical (blue) regions. The granular RSC (areas 29a-c) shows greater connectivity with limbic regions such as the subicular cortex and the antero-dorsal (AD) and antero-ventral (AV) nuclei of the ATN, while the dysgranular RSC (area 30) shows greater connectivity with cortical regions including the parahippocampal region, the posterior parietal cortex, and early visual areas. The connections of the ATN also differ by region, with the AD and AV nuclei showing greater connectivity with limbic areas and the antero-medial (AM) nucleus showing greater connectivity with neocortical regions.

The RSC is a posterior midline structure at the intersection between many limbic and cortical areas involved in spatial memory through its reciprocal connections with the hippocampal formation, ATN, and a network of dorsal-medial cortical regions (Figure 1). The RSC is often described as intermediate or transition cortex because of its position between the 3-layer archicortex of the HPC and the 6-layer neocortex (Vogt, 1976; Vogt and Laureys, 2005). This transition can be seen clearly in the cytoarchitecture of the RSC, with the granular region of the RSC (areas 29a-c) showing greater similarity to hippocampal archicortex and the dysgranular region of the RSC (area 30) showing greater similarity to neocortex. Consistent with this cytoarchitecture, the granular RSC is preferentially connected with limbic regions while the dysgranular RSC is preferentially connected with neocortical regions (Figure 1B). Specifically, the granular RSC shows greater connectivity with the HPC, subicular cortices, and the ATN, while the dysgranular RSC shows greater connectivity with early visual regions, the parietal cortex, and the parahippocampal region (Insausti et al., 1987; Vogt et al., 1987; van Groen and Wyss, 1990, 1992, 2003; Suzuki and Amaral, 1994; Burwell and Amaral, 1998; Morris et al., 1999; Lavenex et al., 2002; Kobayashi and Amaral, 2003, 2007). This pattern of connectivity places the RSC between limbic regions essential for memory formation and cortical regions believed to play an important role in long-term memory storage (Maviel et al., 2004; Frankland and Bontempi, 2005; Aggleton, 2008).

Functional considerations also suggest that the RSC is a critical junction between limbic and cortical regions. The granular RSC forms a limbic circuit with the ATN and the HPC that is essential for many kinds of memory. Damage to any of these three regions reliably disrupts functioning in the remaining two. Electrophysiological research conducted in animals, for example, has demonstrated that ATN lesions performed before training prevent the development of cue-elicited spiking activity in the RSC (Kubota and Gabriel, 1995; Smith et al., 2002), and that lesions of the RSC impair the directional specificity of head direction (HD) cells in the ATN (Clark et al., 2010). Damage to the ATN or RSC also disrupts spatial representations in the HPC: lesions of the ATN degrade the ability of hippocampal place fields to rotate with a shifted visual cue (Calton et al., 2003) and RSC inactivation during navigation causes hippocampal place fields to spontaneously shift to new locations (Cooper and Mizumori, 2001). Likewise, damage to the HPC impairs the normal development of cue-elicited neuronal responses in both the ATN and the RSC (Kang and Gabriel, 1998). Additional studies have shown that lesions of the ATN or the HPC reliably disrupt RSC functions that are essential for memory formation, resulting in a loss of synaptic plasticity and a significant reduction in neural activity, as measured by immediate early gene (IEG) expression (Jenkins et al., 2004; Albasser et al., 2007; Garden et al., 2009). The progression of these disruptions within the RSC is consistent with the idea that the granular RSC is more closely connected with limbic regions, while the dysgranular RSC is more closely connected with neocortical regions. For example, ATN lesions lead to dysregulation of IEG expression in the granular RSC in as little as 1 week, but did not affect the dysgranular RSC until around 1 year (Jenkins et al., 2004; Poirier and Aggleton, 2009). In contrast, lesions of the postrhinal cortex lead to IEG dysregulation in the dysgranular RSC without affecting the granular RSC (Jenkins et al., 2004).

Connections between the dysgranular RSC and other neocortical regions play an important role linking limbic memory areas with spatial and behavioral processing occurring in the dorsal stream. The dorsal stream is a network of cortical structures running from early visual areas to the posterior parietal lobe that have been described as processing “where/how” information (Ungerleider and Mishkin, 1982; Goodale and Milner, 1992). In contrast to the ventral stream, which has been implicated in the distributed representation of object identity (Bussey and Saksida, 2007), the dorsal stream supports coordination between visual inputs and movements (Perenin and Vighetto, 1988), possibly through the simultaneous representation of multiple locations in the visual field (Jackson et al., 2009). The dorsal stream is connected with limbic memory regions such as the HPC via the parieto-medial temporal pathway (Kravitz et al., 2011). This pathway includes bidirectional connections between the posterior parietal lobe and the parahippocampal region that run through the RSC. Combining the dorsal stream and parieto-medial temporal pathway reveals connections from upstream visual areas through posterior parietal regions, the posterior cingulate cortex (PCC) and the RSC, the parahippocampal cortex (PHC) and medial entorhinal cortex, to the HPC downstream. Note that although we describe upstream and downstream components of this processing stream according to the feedforward flow of visual information, there are extensive bi-directional connections throughout. Consistent with their position between limbic memory areas and cortical sensory areas, members of the parieto-temporal pathway contribute to both memory and sensory functions (Epstein and Kanwisher, 1998; Vallar, 1998; Aguirre and D’Esposito, 1999; Vann et al., 2009). However, the contributions of these regions are not identical, and damage to this pathway in humans results in navigation impairments that differ predictably depending on where along the pathway the damage has occurred (Aguirre and D’Esposito, 1999). A broad characterization of these impairments holds that downstream regions participate in more-allocentric types of representation primarily around the time of learning while upstream regions participate in more-egocentric and relatively longer-term representations. Consistent with this, damage downstream from the RSC in the PHC commonly produces allocentric impairments and severe anterograde amnesia, while damage upstream from the RSC in the posterior parietal lobe produces egocentric impairments and a mix of anterograde and retrograde amnesia. Together, these data suggest that spatial memory arises from an extensive network of brain regions including the HPC, ATN, and cortical regions along the dorsal stream. The RSC, in particular, appears to play an essential role uniting regions within this network, and as such is well positioned to contribute to both spatial representation and long-term memory.

## Spatial representation

**Figure 2:**
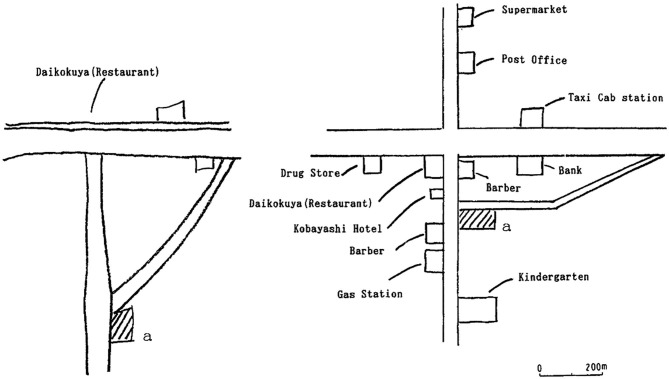
**Impaired map drawing by patient with RSC lesion**. Patients with RSC damage are severely impaired at drawing maps from memory. This patient (Patient 1, Takahashi et al., 1997) was incapable of recalling the locations of buildings in his neighborhood, despite being able to recall their names (left drawing). His wife’s drawing is shown on the right for comparison. Adapted from Takahashi et al. (1997).

Damage to the RSC in humans occurs most frequently following cerebral hemorrhages or tumors in the splenium of the corpus callosum. Patients with RSC damage consistently show navigation impairments and they cannot describe routes between locations or draw maps of the environment despite a preserved sense of familiarity and the ability to recognize individual landmarks and visual scenes (Maguire, 2001; Figure 2). In general, this navigation impairment is referred to as topographical disorientation and can result from damage to any of the structures along the parieto-medial temporal pathway, including the posterior parietal cortex, the RSC, and the PHC (Aguirre and D’Esposito, 1999). However, the particular representational deficit underlying the topographical disorientation differs importantly depending on which region is damaged, with parietal damage (upstream from the RSC) impairing egocentric representations of the environment and parahippocampal damage (downstream from the RSC) impairing more allocentric representations. In the case of RSC damage, topographical disorientation appears to result from a compromised sense of allocentric space, similar to that seen after downstream parahippocampal damage, but particularly involving deficits utilizing landmark information. These impairments manifest as an inability to form or retrieve coherent spatial representations, as evidenced by a deficit in placing landmarks on a map (Takahashi et al., 1997) or localizing oneself within a model of the environment (Katayama et al., 1999).

Some of the strongest evidence for an RSC role in allocentric representation comes from qualitative descriptions of navigation in patients with RSC damage. These reports consistently highlight the inability of these patients to use navigation cues such as landmarks to discern the spatial layout of the environment (Maguire, 2001). For example, Ino and colleagues (Ino et al., 2007) describe the case of a patient who showed a striking impairment in the ability to use landmarks to navigate following a left RSC hemorrhage:

*A 55 year-old right-handed man…who had been working as a taxicab driver in Kyoto City for 10 years, suddenly lost his knowledge of the route to his house while returning home after work. He could recognize buildings and the landscape and therefore understand where he was, but the landmarks that he recognized did not provoke directional information about any other places with respect to those landmarks. …He was unable to determine the direction to familiar destinations within the city with respect to his position at our hospital, although he had often visited this hospital while transporting passengers. He was also unable to describe or draw routes between his home and familiar places in his town, and could not draw the layout of his house*.

Although landmark cues can also support stimulus-response and egocentric navigation strategies, the disruption of cue processing in patients with RSC damage does not appear to impair egocentric representations of space. Instead, egocentric representation is more reliably impaired by damage upstream from the RSC in the parietal lobe (Bisiach and Luzzatti, 1978; Levine et al., 1985; Bisiach et al., 1993; Stark et al., 1996). This distinction can be seen in tasks requiring subjects to point to objects in the testing room from memory or to recall the positions of landmarks that can be seen from their current location—both of which are impaired by parietal damage (Bisiach and Luzzatti, 1978; Levine et al., 1985; Bisiach et al., 1993; Stark et al., 1996), but spared following damage to the RSC (Takahashi et al., 1997). Additional evidence comes from one patient with RSC damage who was incapable of recalling the location of landmarks when questioned about their allocentric position, but who became capable of doing so when cued to adopt an egocentric strategy (i.e., his egocentric frame of reference was preserved; Takahashi et al., 1997, patient 2). When presented with a map of a familiar city but containing only streets and railroad tracks, the patient could not indicate the location of principal city buildings. However, when the examiner indicated a location on the map and told the patient its name, the patient was able to recall the names and positions of buildings within sight of that location. Again, consistent with a role for the parietal lobe in this kind of egocentric representation, the ability to recall landmarks within sight of a given position is often compromised in patients with parietal damage who also suffer from navigation deficits (Bisiach and Luzzatti, 1978; Bisiach et al., 1993). These selective allocentric representational deficits in patients with RSC damage suggest that the RSC has more in common with the downstream parahippocampal region than the upstream parietal lobe in terms of its contribution to spatial representation within the dorsal stream.

Functional magnetic resonance imaging (fMRI) studies in humans have furthered our understanding of allocentric representation in the RSC and the parahippocampal region. These studies have indicated a prominent role for the RSC in processing landmark information and using landmarks to navigate and discern space, while suggesting that the parahippocampal region contributes more to the representation of visual scenes. Adaptation measures have shown that responses in both the RSC and the parahippocampal region are attenuated when subjects are shown different photographs of the same visual scene containing a prominent campus landmark (Morgan et al., 2011). In another study, however, RSC blood oxygen level dependent (BOLD) activation was greater when college students made spatial judgments about campus scenes than when they made simple familiarity judgments, suggesting that the RSC is particularly important for extracting navigation information (Epstein et al., 2007). More recent work has further differentiated these two regions, revealing that the PHC appears to process visual scenes in a way analogous to object identity processing within the ventral stream, while the RSC is more sensitive to the *layout* of a navigable scene. For example, when participants were shown a visual scene followed by either the same scene again or its mirror reversal, the RSC showed attenuation to the same scene, but not to the mirror reversal (which altered the relationships between elements in the portrayed physical space), while the PHC showed attenuation in both conditions (Dilks et al., 2011). Additionally, RSC activity is closely related to the permanence of landmarks, a feature that is essential to building a long-term representation of the layout of the environment. When subjects were presented with isolated images of many different landmark objects, RSC activation increased specifically with the permanence of objects, while parahippocampal activity increased with a wide range of attributes (Auger et al., 2012; Auger and Maguire, 2013). Importantly, the ability to identify permanence was closely related to navigation ability, as good navigators made more reliable decisions about object permanence than did poor navigators, and showed greater RSC activation (but not greater parahippocampal activation) when viewing permanent objects (e.g., a fire hydrant). Together, these results suggest that while the RSC shares many features of allocentric representation with the parahippocampal region, the RSC seems particularly sensitive to the permanent features such as landmarks and other navigation cues that define the layout of the environment.

**Figure 3:**
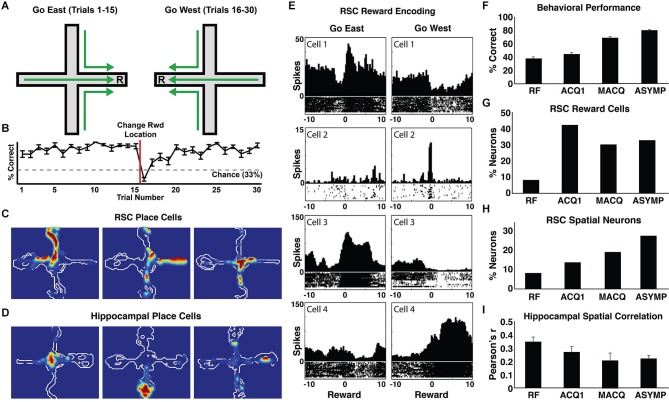
**Neural coding by RSC and hippocampal neurons during navigation**. **(A)** A schematic of the blocked alternation task from Smith et al. (2012). Rats navigated to one location for a reward during the first 15 trials of every session (east arm), and navigated to a different location (on the same maze) during the second 15 trials (west arm). **(B)** Rats used the reward location as a navigation cue. Line graph shows average percent correct on each trial during asymptotic performance of the blocked alternation task. Rats employed a win-stay strategy, returning to the location that was rewarded on the previous trial. After discovering that the reward location had changed on trial 16, the rats began navigating to the new reward location. **(C)** Examples of RSC place cells and **(D)** hippocampal place cells. **(E)** Examples of RSC location-specific reward responses. Each pair of perievent time histograms shows the spiking activity of a single neuron, with the left and right histograms showing firing during the 10 s before and after the go east and go west rewards. RSC neurons occasionally showed punctuate firing at the instant of one reward (cells 1–2). Other RSC neurons showed sustained elevated firing while the rat consumed the reward in one location, and decreased firing during the other reward (cells 3–4). **(F)** Navigation performance improved with training. The average percentage of trials in which the rats made the correct arm entry is shown for random foraging pretraining (RF), the first training session (ACQ1), the session half way through training (middle acquisition, MACQ), and asymptotic performance (ASYMP). **(G)** The proportion of RSC neurons that differentially encoded the two reward locations increased dramatically on the first day of training (when the reward locations became useful navigation cues) and then remained high throughout the rest of training. **(H)** The proportion of RSC neurons with place fields increased steadily with training. **(I)** Although the proportion of hippocampal neurons with place fields remained the same throughout training (not shown), the activity of these neurons became more distinct in the Go East and Go West conditions as the rats learned the task (average spatial correlations, Pearson’s *r*). Adapted from Smith et al. (2012).

A growing body of work with rodents has also implicated the RSC in allocentric navigation. Lesions of the RSC impair performance on a wide range of navigation tasks requiring the use of distal cues, including the Morris water maze (Vann and Aggleton, 2002; Harker and Whishaw, 2004; Pothuizen et al., 2010), radial arm maze (Vann and Aggleton, 2002; Keene and Bucci, 2009), and t-maze alternation tasks (Pothuizen et al., 2010). RSC neurons also exhibit many of the firing patterns seen in typical neurophysiological studies of spatial navigation, including spatially localized firing (i.e., place cells) and firing modulated by head direction. Approximately 25% of RSC neurons have place fields and 10% show firing correlated with HD (Chen et al., 1994a,b; Cho and Sharp, 2001). While the unique features of these cells have yet to be thoroughly characterized, RSC place fields are generally larger than their counterparts in the HPC (about three times as large in our studies; compare Figures 3C–D; Smith et al., 2012), and have much higher background firing rates than hippocampal neurons. Likewise, HD cells in the RSC have a narrower tuning width than HD cells recorded elsewhere (Cho and Sharp, 2001). The RSC also shows a clear theta oscillation in its local field potential that may be involved in navigation. In the HPC, the theta oscillation is thought to play a critical role in organizing spiking activity in a manner that enables neural plasticity (Skaggs et al., 1996; Buzsáki, 2005; van der Meer and Redish, 2011; Brandon et al., 2013) and provides a mechanism for coordinating spiking activity between the HPC and various cortical regions (Jones and Wilson, 2005; Sirota et al., 2008). The details of theta interactions between the HPC and RSC are not well understood, with some findings indicating that hippocampal lesions eliminate cue-elicited theta in the RSC, and others suggesting that some aspects of RSC theta may be independent of the HPC (Borst et al., 1987; Talk et al., 2004). Nonetheless, theta in the RSC is prominent during locomotion, as is seen in the HPC, and RSC theta is frequently phase locked with hippocampal theta (Leung and Borst, 1987; Young and McNaughton, 2009). Additionally, theta frequency and power decrease in both regions as rats explore a new environment, suggesting a role in forming spatial representations (Young and McNaughton, 2009).

Similar to the work in humans described above, our neurophysiological work with rodents has shown that RSC neurons are also highly sensitive to navigation cues. In our experiments (Smith and Mizumori, 2006b; Smith et al., 2012), rats were trained in a blocked alternation task in which they followed a “go east” rule (regardless of the start position, go to the end of the east arm of the maze for reward) during the first half of each session and a “go west” rule during the second half (Figure 3A). In this task, the rats use a “win-stay” strategy wherein they start each session by going to the east arm and they continue as long as rewards are found there. They only switch to approaching the west arm after discovering (on trial 16) that the east arm is no longer rewarded (Figure 3B). Because of this, the reward location is a critically important navigation cue. On each trial, this cue informs the rat about which rule—go east or go west—is in effect and, therefore, where the reward can be found on the next trial. RSC neurons were remarkably sensitive to this critical cue, with 30–40% of granular RSC neurons exhibiting significant responses that differentiated the east and west reward locations. Although some neurons exhibited very low background firing rates with a sharp burst of spikes at the reward location (e.g., Figure 3E, cells 1–2), most RSC neurons exhibited “on/off” responses involving increased firing at one reward location and decreased firing at the other location (e.g., Figure 3E, cells 3–4), perhaps serving to maximally differentiate the two reward locations. Consistent with this sensitivity to navigation cues, one of the early reports of RSC spatial responses (Chen et al., 1994a) described a neuron that exhibited its highest firing rate when the rat traversed toward a white cue card, regardless of the rat’s location. Thus, at the level of individual neurons and regional BOLD signals, the RSC is highly responsive to navigation cues.

The RSC also makes a critical contribution to allocentric navigation by representing the spatial relationships among navigation cues. As described above, humans with RSC damage are not impaired at recognizing familiar landmarks, but are nonetheless impaired at using them to navigate (Takahashi et al., 1997). Similarly, rats with RSC damage remain capable of detecting novel objects, but are specifically impaired at detecting changes to the spatial configuration of objects (Parron and Save, 2004; see also Vann and Aggleton, 2002; but see Haijima and Ichitani, 2012). This is in contrast to lesions of the perirhinal cortex in the ventral stream, which impair the ability to detect novel objects, but do not impair spatial navigation (Winters et al., 2004). An impaired ability to represent the relationships between navigation cues likely underlies the stark inability of rats with RSC lesions to navigate on a rotated maze, as this manipulation brings local and distal cues into conflict, requiring an on-the-fly updating of the spatial relationships among these navigation cues (Pothuizen et al., 2008). Consistent with these findings, many RSC neurons respond to combinations of cues, location, and directional heading, sometimes exhibiting surprising selectivity (Cho and Sharp, 2001). For example, one RSC neuron described by Cho and Sharp (2001) fired preferentially when the rat was near the arena wall, turning left toward the center of the arena. This conjunctive coding is similar to that seen in hippocampal neurons, which frequently respond selectively to combinations of locations, objects, and events—a mechanism thought to be a hallmark of hippocampal relational memory functions (e.g., Komorowski et al., 2009). Although these cells have not been thoroughly characterized in the RSC, it is possible that they encode the conjunctions of spatial position, navigation cues, and navigation behaviors necessary to create an allocentric representation of the environment. Indeed, the location-specific reward response mentioned above (Figure 3E) is a particularly clear example of conjunctive coding underlying the representation of a navigation cue. Together, these findings suggest that the critical contribution of the RSC to allocentric navigation may be the representation of spatial relationships between stable features of the environment that serve as important navigation cues, such as landmarks.

## Context

RSC contributions to spatial cognition are likely related to its role in support of context representations. The context is traditionally defined as the set of continuously present background cues and is closely related to the idea of spatial representations (Nadel et al., 1985; Mizumori et al., 2007). Many of the brain regions known to be involved in spatial navigation also play a prominent role in processing contextual information. For example, the HPC is involved in a wide range of contextual memory tasks, such as contextual fear conditioning (Kim and Fanselow, 1992; Phillips and Ledoux, 1992) and the ability to match a learned behavior with the appropriate context (Good and Honey, 1991; Penick and Solomon, 1991; Smith et al., 2004), and hippocampal neurons are highly sensitive to contextual variables (e.g., Anderson and Jeffery, 2003). The RSC is also importantly involved in contextual memory processes. Lesions of the RSC impair contextual fear conditioning (Keene and Bucci, 2008a,c), and RSC neurons exhibit context specific patterns of spiking activity during learning (Freeman et al., 1997; Smith et al., 2004). RSC projections to the postrhinal cortex (one route for RSC input to the HPC) show IEG activation during contextual fear conditioning and disruption of this input impairs contextual learning (Bucci et al., 2002; Robinson et al., 2012). Interestingly, lesions upstream from the RSC in the posterior parietal cortex do not impair contextual fear memory (Keene and Bucci, 2008a), suggesting that the RSC may be the first region along the dorsal stream that is necessary for this kind of context processing.

The RSC also supports the association between objects or behaviors and the context in which they occur. For example, it is well known that objects are recognized more quickly and accurately when they are situated within a typical context than when they are in an unusual context (Palmer, 1975; Biederman et al., 1982), suggesting that object representations are closely associated with representations of the context. Consistent with this idea, the RSC shows greater BOLD activation when subjects view objects with strong contextual associations (e.g., a beach shell) than when they view objects that are not strongly associated with a particular context (e.g., a camera; Bar and Aminoff, 2003; Bar, 2004). The RSC may also support associations between behaviors and the context. RSC neurons exhibit distinctive patterns of cue-evoked spiking activity during instrumental discrimination that are specific to a particular context (Freeman et al., 1997; Smith et al., 2004), and damage to the RSC impairs the ability to respond selectively to a cue that was previously found in the current context (Nelson et al., 2014). We have recently shown that two structures that are anatomically interconnected with the RSC—the HPC and anterior thalamus—are also necessary for using contextual information to disambiguate the meaning of cues (Butterly et al., 2012; Law and Smith, 2012), suggesting that associations between behaviors, cues, and the context may result from interactions between these three structures.

The RSC also participates in the more general use of contextual information to support perceptual processing and memory formation (Bar, 2004; Bar et al., 2007). For example, RSC processing appears to support the boundary extension phenomenon (Park et al., 2007; but see Chadwick et al., 2013)—a type of false memory whereby observers report having seen information that they extrapolated beyond the actual boundaries of the scene (Intraub et al., 1996). Another example is the critical role played by long-term chess memory in new memory formation. Chess experts possess long-term knowledge about the spatial and functional relationships among chess pieces and show greater memory for chess-typical arrangements of these pieces than random arrangements (Chase and Simon, 1973). In a recent fMRI study, expert and novice chess players identified the number of threats, in which one piece is positioned to attack an opposing piece, present within either random or chess-typical arrangements of chess pieces (Bilalić et al., 2012). BOLD activity in the RSC was more sensitive to chess-typical arrangements than to random arrangements exclusively among the expert chess players, suggesting that the memory benefits afforded by chess expertise may be related to long-term spatial and contextual information processed in the RSC. Context processing by the RSC also appears to support the use of long-term spatial expertise to rapidly acquire and consolidate new memories. In a study of spatial schemas in rats, increased IEG expression in the RSC was observed exclusively when long-term spatial memory was used to rapidly acquire new spatial information (Tse et al., 2011). Interestingly, rats were only capable of this rapid memory acquisition when the context was consistent with their relevant long-term spatial memories (i.e., rats only showed the memory benefit when new learning occurred in the same room that was used for training), suggesting that RSC context processing supports this kind of memory acquisition (Tse et al., 2007).

As in spatial representation, where the RSC processes the relationships among landmarks and other navigational cues, the role of the RSC in context processing may also involve encoding cue relationships. In order to create a coherent context representation, subjects must link a variety of multimodal cues—including non-spatial cues—and their relationships into a coherent representation (Balsam and Tomie, 1985; Sutherland and Rudy, 1989; Rudy and Sutherland, 1995). Consistent with this, RSC lesions that impair contextual fear conditioning have no effect on learning to fear a phasic cue, suggesting a specific role in representing the associations between the many cues that comprise the context (Keene and Bucci, 2008c). Similarly, the RSC is needed when subjects must use compound stimuli, such as a tone-light combination, to guide behavior (Keene and Bucci, 2008b; Robinson et al., 2011), and is also necessary for creating associative links between stimuli simply because they co-occur (Robinson et al., 2011). Related work with humans has also shown increases in RSC BOLD activity in response to arrays of objects that typically appear together within the same context (e.g., office items), including contexts that are not defined by a particular location, such as “cosmetics” or “birthday” (Bar and Aminoff, 2003), suggesting that this RSC activity is related to the associations formed between these objects over many paired exposures.

This evidence for RSC involvement in non-spatial aspects of context processing argues against the idea that RSC processing is limited to spatial cognition. Indeed, the RSC is critically involved in a number of tasks that have no obvious spatial component. For example, an extensive literature has implicated the RSC in instrumental discrimination learning wherein subjects learn that one auditory tone signals a foot shock that can be avoided with a locomotor response while another tone signals safety (for review see Gabriel, 1993; see also Smith et al., 2002). The RSC is also necessary for other conditioning tasks involving multiple cues, including conditional visual discrimination (Bussey et al., 1996), and compound (Keene and Bucci, 2008b) and serial feature negative discrimination (Robinson et al., 2011). A common feature of these tasks is that subjects can only perform optimally if they are able to form the appropriate associations among multiple cues and outcomes, suggesting that the processing of complex cue associations may be the basic RSC function underlying both spatial cognition and context processing.

## Episodic memory and amnesia

The relationship between spatial memory and many other forms of memory processing depends critically upon the role of contextual information (for reviews see Smith and Mizumori, 2006a; Smith and Bulkin, 2014). Indeed, spatial and contextual memory are often described as examples of the same relational memory processing thought to support episodic memory, as all of these processes rely on representations of objects and object relationships (Eichenbaum et al., 1999). Several lines of evidence suggest that the RSC is also involved in this kind of memory representation. For example, RSC damage causes amnesia for episodic memory that is similar to that seen after temporal lobe damage, including profound anterograde amnesia and temporally graded retrograde amnesia. The most famous case of RSC amnesia was described in patient T.R., who suffered a left RSC hemorrhage at the age of 39 (Valenstein et al., 1987; Bowers et al., 1988). T.R. experienced severe anterograde amnesia for declarative information and retrograde amnesia for events occurring in the 4 years preceding the injury, resulting in the loss of memory for the birth of his second child, a job change, and a recent relocation. Other patients with RSC lesions have shown even greater retrograde amnesia (e.g., ≥20 years in patient A.P.; Gainotti et al., 1998). The similarity between RSC amnesia and other amnesic syndromes, combined with recent findings that hippocampal or ATN damage disrupts functioning in the RSC (Jenkins et al., 2004; Albasser et al., 2007; Garden et al., 2009; Poirier and Aggleton, 2009), has led some authors to suggest that deficits in memory processing resulting from temporal lobe or diencephalic damage may be due, in part, to RSC dysfunction (Aggleton, 2008). It is noteworthy, however, that not all patients with RSC damage show impaired episodic memory. Unilateral lesions of the RSC can selectively produce either pure topographical disorientation (e.g., Takahashi et al., 1997) or occasionally memory impairments without topographical deficits (e.g., Oka et al., 2003), suggesting that spatial and episodic functions in the human RSC may be lateralized, as is seen in the human HPC (Maguire, 2001).

**Figure 4:**
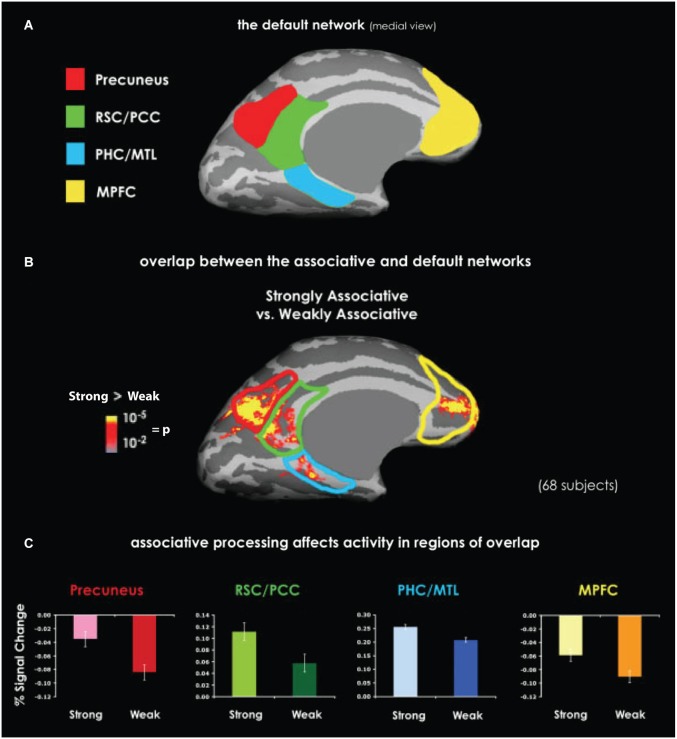
**Overlapping activation between the default network and the context association network**. **(A)** Medial view of the default network. The labeled regions, including the RSC, are those that typically show greater BOLD activation during inter-trial rest than during task performance. **(B)** An activation map shows the difference between perceiving highly contextual objects (e.g., a shopping cart) and weakly contextual objects (e.g., a rope). Data were obtained by averaging together six similar experiments. The superimposed outlines of default network areas demonstrate the overlap between context processing and the default network. **(C)** Activity related to context processing in the regions of overlap with the default network, as manifested by percent of signal change. In each of these regions, highly contextual objects elicited either stronger positive or less negative activation compared with weakly contextual objects. Abbreviations: retrosplenial cortex (RSC), posterior cingulate cortex (PCC), parahippocampal cortex (PHC), medial temporal lobe (MTL), medial prefrontal cortex (MPFC). Adapted from Bar et al. (2007).

Additional evidence for an RSC role in episodic memory processes comes from studies of the default network, a group of cortical structures, including the RSC, the parahippocampal cortex, and the posterior parietal cortex, originally identified by their higher levels of BOLD activation during inter-trial rest periods than during goal-directed behavior (Raichle et al., 2001). The observation that these regions are often less active during task performance than during inter-trial rest has led some authors to describe them as comprising a “task-negative” network (e.g., Fox et al., 2005). Contrary to this, recent data suggest that default network activity reflects internally driven memory processing (Mason et al., 2007; Buckner et al., 2008; Spreng, 2012). The default network is active during a range of processes relevant to episodic memory, including self-projection (Buckner and Carroll, 2007), constructive memory (Hassabis and Maguire, 2007), and autobiographical memory (Spreng et al., 2009). Furthermore, naturally occurring conditions that compromise the default network, including Alzheimer’s disease and normal ageing, are well known to impair episodic memory (Lustig et al., 2003; Buckner et al., 2005). Among the members of the default network, meta-analyses have consistently identified the RSC as one of the most reliably activated (Shulman et al., 1997; Buckner et al., 2008; Spreng et al., 2009). The precise contribution of the RSC to default network function is not known. However, many default network tasks have a prominent contextual component. Indeed, many of the cortical regions commonly involved in default network functions are also involved in context processing (Bar et al., 2007; Figure 4), opening the possibility that context representations are a key contribution of this network to episodic memory.

## Learning and consolidation

In addition to the general roles in spatial, contextual, and episodic memory processing described above, a growing literature suggests that the RSC plays a key role in memory acquisition and consolidation through its interactions with the HPC. Damage to the RSC in humans results in both anterograde and retrograde amnesia, impairing new spatial learning and navigation within environments learned long before the RSC was damaged (Obi et al., 1992; Takahashi et al., 1997). Consistent with this, the neural correlates of cue and allocentric representation develop in the RSC over the course of learning. In our studies of blocked alternation learning (Figure 3; Smith et al., 2012), we examined the development of RSC neuronal responses as the rats learned to navigate and, as described above, found that RSC neurons exhibited place fields and highly selective responses to rewards in particular locations. Rats showed a steady increase in navigation accuracy over the course of training, which took about seven training sessions (Figure 3F). Interestingly, RSC place fields and reward responses showed different patterns of development over learning. As mentioned above, because the rats used a win-stay strategy, the reward location was a critical navigational cue that informed the rat about upcoming reward locations. Reward responses developed very rapidly in the RSC, with more than 40% of RSC neurons exhibiting east- or west-selective reward responses on the first day of training, before the rats exhibited significant behavioral evidence of learning. As learning continued, the number of neurons encoding the reward locations remained high, with about 30% of RSC neurons showing differential reward responses (Figure 3G). In contrast, RSC place fields developed more slowly. During preliminary training sessions, which involved foraging for randomly placed rewards without the go east or go west contingencies in effect, less than 10% of RSC neurons exhibited place fields. However, this percentage increased steadily throughout training until about 28% had place fields during asymptotic performance (Figure 3H). This stands in contrast to hippocampal place fields, which develop rapidly and remain stable thereafter (Thompson and Best, 1990; Frank et al., 2004; Smith et al., 2012; but see Mankin et al., 2012). Additionally, while hippocampal place fields in our study came to differentiate the go east and go west conditions as the rats learned (i.e., hippocampal place cells developed different place field locations for each condition; Figure 3I), RSC place fields were similar in each condition, consistent with the idea that the RSC represents the stable features of the environment independent of the current navigation strategy. This evidence suggests that the RSC may immediately encode prominent cues in the environment and then slowly develop a broader representation over the course of learning.

Other recent evidence additionally suggests that the RSC may store long-term spatial and contextual memories as a result of a hippocampal-dependent systems consolidation process. For example, inhibitory avoidance learning, wherein rats learn to avoid a shock zone within an apparatus, initially requires both the HPC and the RSC (Zanatta et al., 1997; Katche et al., 2013a). After about 14 days, the memory is no longer hippocampal dependent (Izquierdo and Medina, 1997), although it remains dependent on the RSC (Katche et al., 2013b). The formation of a long-term memory in the RSC in this task appears to depend on interactions between the HPC and the RSC occurring around 12 h after learning, when waves of IEG activation and protein synthesis occur in both regions that are necessary for the development of long-term (but not short-term) memory (Katche et al., 2010, 2013a). A number of other studies have also shown that spatial and contextual memories continue to rely on the RSC after they have become hippocampal independent, leading some authors to suggest that critical elements of these memories are permanently stored in the RSC (Maviel et al., 2004; Gusev and Gubin, 2010; Corcoran et al., 2011; but see Hart et al., 1997). The development of RSC neural responses also suggests a role for the RSC in the long-term memory consolidation. In a contextual version of a cue discrimination task (Gabriel, 1993), distinctive patterns of context-specific cue-evoked spiking activity develop slowly in the RSC as subjects learn to discriminate two cues with unique contingencies in different contexts, suggesting that they represent the association of the learned discrimination with the context (Freeman et al., 1997; Smith et al., 2004). Furthermore, when subjects were given a 2-day gap in discrimination training, RSC firing patterns continued to evolve and behavioral performance improved, even without additional training trials, suggesting that RSC-dependent processes supported memory consolidation during that period (Freeman and Gabriel, 1999). Consistent with the idea that the development of long-term memory in the RSC depends on interactions with the HPC, disrupting hippocampal input to the RSC in this task degrades the development of context-dependent neuronal activity patterns and impairs the ability to match a learned discrimination with the appropriate context (Smith et al., 2004). Recent evidence has also implicated the RSC in a process by which the HPC rapidly incorporates novel spatial information into a long-term spatial representation in cortex (Day et al., 2003; Tse et al., 2007, 2011). In this work, rats developed long-term memories for food locations, and the presence of these memories enabled the rats to rapidly learn and consolidate new spatial information at an accelerated rate that is consistent with systems reconsolidation (e.g., Debiec et al., 2002). While hippocampal IEG activation increased immediately following any type of learning, RSC IEG activation increased only when long-term spatial memories facilitated new acquisition, consistent with the idea that the rapid learning resulted from the consolidation of novel information into a long-term spatial representation stored in the RSC (Tse et al., 2011).

**Figure 5:**
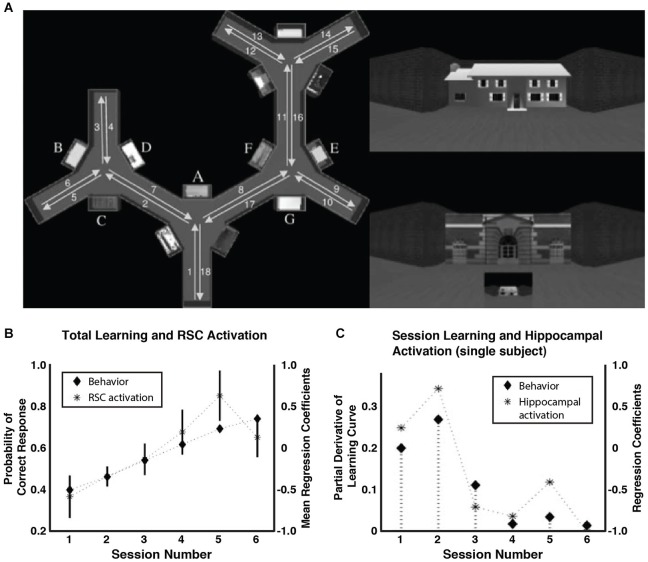
**RSC and hippocampal activation during spatial navigation learning**. **(A)** Participants received first-person tours of a virtual maze. On the left is a bird’s-eye-view diagram of the maze (not shown to participants) showing the locations of the landmarks and the route of the tour. In the upper right is an example of the first-person view of a landmark. The lower right shows an example test question. Participants indicated by button press the relative position of the small building, imagining that they were standing in front of the large building. **(B)** RSC BOLD activation increased along with behavioral accuracy over the course of training. **(C)** In contrast, hippocampal activation was specifically correlated with the *slope* of the learning curve (data from one participant shown). Adapted from Wolbers and Buchel (2005).

Evidence from fMRI studies of human subjects has also suggested that a hippocampal process supports the development of memory representations in the RSC. Several fMRI studies have shown increases in BOLD activation in the RSC during spatial learning (Aguirre et al., 1996; Grön et al., 2000; Iaria et al., 2007). In one such study (Wolbers and Buchel, 2005; Figure 5), subjects received repeated first-person point of view tours of a virtual maze lined with visual landmarks. After each tour, participants were asked to imagine that they were facing a particular landmark and to indicate the relative position of a second landmark. With training, most of the participants became as accurate at judging the relative positions of landmarks located far apart on the maze as they were at judging pairs of landmarks encountered in sequence along the tour, suggesting that they had developed an allocentric representation of the maze. RSC activity increased as these participants learned the task, consistent with its role in the development of an allocentric representation (Figure 5B). Interestingly, hippocampal activity did not increase over the course of training, but was instead correlated with the *slope* of the learning curve (Figure 5C). That is, the degree of hippocampal activation predicted the amount of learning occurring in a given session and, as a consequence, hippocampal activation declined with training as the subjects reached asymptote. This suggests that hippocampal-dependent processes support the slow-developing allocentric representation in the RSC, perhaps by incorporating novel information into the long-term memory trace—albeit over the relatively short timeline of a single training session. One possibility is that information acquired during learning is replayed by the hippocampal system during offline periods, similar to work in rats showing hippocampal replay of place cell activity during rest periods (Wilson and McNaughton, 1994; Carr et al., 2011), and that this replay supports memory consolidation and learning. Consistent with this, recent evidence has shown that reactivation processes occurring during awake, post-learning periods in the RSC and the medial temporal lobe (MTL) are related to later memory performance on a visual paired-associate test (Staresina et al., 2013).

## A working model of RSC-hippocampal interaction

**Figure 6:**
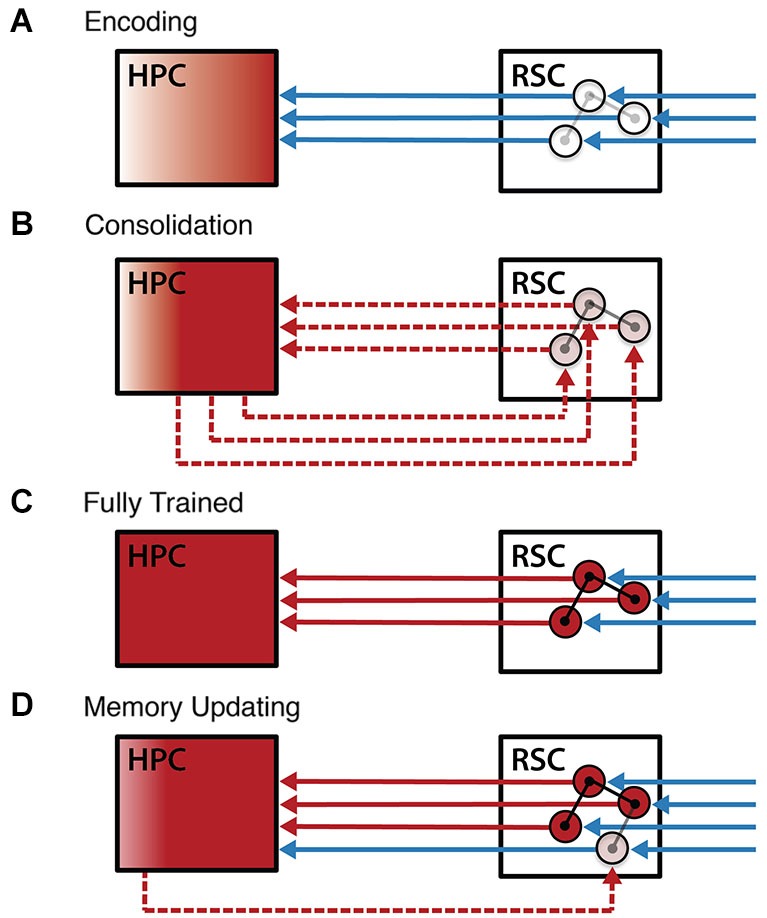
**Working model of RSC-hippocampal interaction during learning**. A model of RSC-hippocampal interactions over the course of learning. **(A)** Encoding in naïve animals is characterized by feedforward sensory information (blue lines) driving plasticity in both the cortex and the HPC. Plasticity in the RSC (circles and their connections) includes the early formation of cue-related activity, while plasticity in the HPC leads to the formation of a stable hippocampal memory representation (red). **(B)** After the learning event, interactions between the RSC and the HPC (dashed red lines) consolidate memories into a more stable form. **(C)** In the fully trained subject, feedforward sensory input activates the consolidated RSC memory representation, which, in turn, activates the corresponding representation in the HPC. **(D)** Coordinated activity in the RSC and HPC enables memory updating, whereby the detection of novelty by the HPC initiates rapid consolidation of this new information into the existing cortical memory trace. Abbreviations: retrosplenial cortex (RSC), hippocampus (HPC).

The data reviewed here indicate that the RSC plays a critical role in spatial, contextual, and relational memory, and that interactions between the RSC and the HPC are essential for normal memory processing. These data highlight the dual role of the RSC as both an input structure to the HPC that contributes importantly to the processing of cues related to navigation and context, and as a target of hippocampal output during systems consolidation. It is likely that these two roles are closely related, with previously consolidated long-term memories augmenting the processing of feedforward sensory input by the RSC (Figure 6). Specifically, we have presented evidence that: (1) RSC input to the HPC is critical for the normal encoding of context representations; (2) consolidation processes begin to establish memory traces in the cortex (McClelland et al., 1995), including the RSC, after encoding (3) the RSC provides input to the HPC that is crucial for identifying the active context in fully trained subjects; and (4) this RSC input allows the HPC to incorporate novel input into the appropriate memory trace. Supporting these assertions, lesions of the RSC during the learning process impair memory acquisition (Valenstein et al., 1987; Ino et al., 2007; Keene and Bucci, 2008c; Danker and Anderson, 2010; Corcoran et al., 2011; Katche et al., 2013a), possibly by disrupting hippocampal processing (Cooper and Mizumori, 2001). RSC damage also impairs late-stage learning processes (Gabriel, 1993; Bussey et al., 1996) and long-term memory (Valenstein et al., 1987; Takahashi et al., 1997; Gainotti et al., 1998; Danker and Anderson, 2010; Katche et al., 2013a). The formation of long-term memory in the RSC depends on hippocampal-dependent processes (Maviel et al., 2004; Wolbers and Buchel, 2005; Katche et al., 2013a; Staresina et al., 2013). RSC-hippocampal interactions continue to be important for learning in the fully trained subject, as RSC input supports ongoing hippocampal processing (Cooper and Mizumori, 2001) and interplay between these regions facilitates the updating of existing memories (Tse et al., 2007, 2011). The factors that influence the direction of information flow and the interplay between new learning, consolidation, and memory updating in the RSC and HPC remain poorly understood and future studies will need to simultaneously monitor processing in both regions before a truly detailed account can be formulated.

## Concluding remarks

The RSC plays a prominent role in spatial and contextual memory processes, possibly owing to a fundamental role in processing cues and the relationships between them. Moreover, we suggest that the RSC can best be understood as a critical node within a larger circuit that mediates the related functions of spatial cognition, context representation, and episodic memory. Indeed, the RSC is positioned at the intersection of the neocortical dorsal stream and the limbic memory circuit. We suggest a general view of the dorsal stream as a functional gradient spanning from egocentric representations in upstream regions to allocentric representations in downstream regions. Within this framework, the RSC plays a dual role as both a processing node supporting the feedforward transmission of sensory-perceptual information to the limbic memory circuit and as a target of consolidation for long-term memory. Additional research will be needed to provide a detailed account of RSC spatial and contextual representations and an understanding of the dynamic processes that control information flow between the RSC and the HPC. In particular, a greater understanding of the interactions between the limbic memory circuit and the neocortex will help to clarify the relationships between sensory processing, memory formation and consolidation, and long-term memory representations.

## Conflict of interest statement

The authors declare that the research was conducted in the absence of any commercial or financial relationships that could be construed as a potential conflict of interest.
